# Ant queens increase their reproductive efforts after pathogen infection

**DOI:** 10.1098/rsos.170547

**Published:** 2017-07-05

**Authors:** Julia Giehr, Anna V. Grasse, Sylvia Cremer, Jürgen Heinze, Alexandra Schrempf

**Affiliations:** 1 Zoology/Evolutionary Biology, University of Regensburg, 93053 Regensburg, Germany; 2 IST Austria (Institute of Science and Technology Austria), Am Campus 1, 3400 Klosterneuburg, Austria

**Keywords:** terminal investment, social insect, infection, reproduction

## Abstract

Infections with potentially lethal pathogens may negatively affect an individual's lifespan and decrease its reproductive value. The terminal investment hypothesis predicts that individuals faced with a reduced survival should invest more into reproduction instead of maintenance and growth. Several studies suggest that individuals are indeed able to estimate their body condition and to increase their reproductive effort with approaching death, while other studies gave ambiguous results. We investigate whether queens of a perennial social insect (ant) are able to boost their reproduction following infection with an obligate killing pathogen. Social insect queens are special with regard to reproduction and aging, as they outlive conspecific non-reproductive workers. Moreover, in the ant *Cardiocondyla obscurior*, fecundity increases with queen age. However, it remained unclear whether this reflects negative reproductive senescence or terminal investment in response to approaching death. Here, we test whether queens of *C. obscurior* react to infection with the entomopathogenic fungus *Metarhizium brunneum* by an increased egg-laying rate. We show that a fungal infection triggers a reinforced investment in reproduction in queens. This adjustment of the reproductive rate by ant queens is consistent with predictions of the terminal investment hypothesis and is reported for the first time in a social insect.

## 1. Introduction

Life-history theory predicts that organisms increase their investment into current reproduction when their residual reproductive value decreases with age and approaching death [1–4]. Documenting this ‘terminal investment’ [4] has often been difficult as aging is usually associated with a physical decline (senescence) and limited resource availability. Both may obscure increased reproductive efforts at higher age [2,5–7]. While some studies have documented terminal investment (e.g. in birds [8,9], amphibians [10] and solitary insects [11–15]), others gave inconclusive [1] or even contradicting results (e.g. in fishes [16] and mammals [2]).

Infection with pathogens has been used to test whether animals can react to a decline of physical condition independent of chronological age. In some species, infections indeed resulted in reinforced investment in reproduction (e.g. [9,10,17]) while in others the activation of the immune system was associated with a decrease in reproduction (e.g. lizards [18]). Here, we use a social insect to investigate whether ant queens are capable of reacting to infection by an increased egg-laying rate, as expected from the terminal investment hypothesis.

Queens of perennial social insects (ants, bees, termites) are exceptional with regard to life-history trade-offs and senescence. First, they show an extreme extension of lifespan compared to most solitary insects [19,20]. Second, queens and also reproductive workers live longer than their non-reproductive worker nest-mates, suggesting the absence of a trade-off between reproduction and lifespan on the level of the individual [21–23]. In *Cardiocondyla* ant queens*,* weekly egg-laying rates were shown to be positively associated with longevity and to gradually increase with age [24–26], suggesting negative senescence. Here we show that, beyond that, infection with an entomopathogenic fungus triggers a boost in reproduction in queens of *Cardiocondyla obscurior*, independent of chronological age*.* Our data in a social insect therefore are in line with the terminal investment hypothesis.

## 2. Methods

*Cardiocondyla obscurior* lives in small colonies with less than 100 sterile workers and one or a few queens [27,28], which have a mean lifespan of 26 weeks [29]. We used large laboratory stock colonies that had been kept in the laboratory for several years to set up experimental colonies, each with one queen pupa, one pupa of a wingless male, and 20 workers. Worker number was kept constant over time by adding worker pupae from stock colonies or removing surplus workers. Sexuals mated after hatching and all eggs produced by the mated queen were counted at least twice per week. Queen pupae that developed from the brood were removed to avoid hatching of a second queen. Colonies were reared in incubators with a 12 h 28°C/12 h 23°C cycle and fed twice per week with cockroaches or fruit flies and honey. The queens were about nine weeks old when the treatment started (high infection: median age 65 days, Q1 60.8, Q3: 69.3; Low infection: median age 67 days, Q1 60.0, Q3: 70.0; Control: median age 63 days, Q1 54.8, Q3: 71.3).

To investigate whether queens increase their reproductive efforts in response to a pathogen, we exposed some of the queens to conidiospores of the entomopathogenic fungus, *Metarhizium brunneum. Metarhizium brunneum* is an obligate-killing pathogen, requiring host death for the completion of its life cycle [30–32]. *Metarhizium brunneum* penetrates the host cuticle within 48 hours after exposure and thereafter grows hyphae in the insect body [33]. Fungal growth and toxins released by the fungus after infection lead to host death, followed by outgrowth of a new generation of infectious conidiospores [34]. It is known from other ants that exposure does not always lead to lethal high-level infections, but can also result in asymptomatic low-level infections [35]. To increase the proportion of queens that developed a lethal infection, we performed a preliminary test that revealed that dipping ant queens in a spore suspension with a concentration of 5 × 10^7^ spores ml^−1^ leads to 53.3% of *C. obscurior* queens developing lethal infections within 35 days after exposure, which is required to test the terminal investment hypothesis. Queens from 43 successfully established experimental colonies were subjected to three different treatments: (i) treatment with a 5 × 10^7^ spores ml^−1^ *Metarhizium brunneum* spore suspension (strain Ma 275; KVL 03-143; as in [36], in 0.05% Triton X; *n* = 31), (ii) treatment with 0.05% Triton X solution (*n* = 6) and (iii) an untreated control group (*n* = 6). Queens of the 1st and 2nd group were grasped with forceps and completely dipped into the *M. brunneum* spore suspension or the 0.05% Triton X solution, respectively, for 15 seconds (in a small bowl, approx. 2 ml) or until they had become immobile. Excessive liquid was removed from the queen's surface by placing the queen on filter paper directly following the dipping procedure. Treated queens were isolated from their colony for 30 h to avoid grooming and spore removal by workers. Queens of the third group were removed from the colonies to control for the effects of isolation. Preliminary data had shown that many queens die within a few hours after treatment with spore suspension, hence the sample size for this group was much larger than that for the two control groups 2 (‘Triton X’) and 3 (‘Untreated’), in which survival rate was high.

After the return of the queens, colonies were kept at room temperature (approx. 23°C). All eggs were counted daily and dead queens were removed and frozen to determine their pathogen load by quantitative real-time PCR on the fungal ITS2 rRNA gene region [37,38]. Queens that were still alive after 24 days were frozen for the detection of their pathogen load and the verification that control queens were not infected, respectively. Prior to DNA extraction individual ants were homogenized using a sterile micropestle. To ensure rupture of the spores the samples were additionally homogenized with acid-washed glass beads (425–600 µm; Sigma-Aldrich) using a TissueLyzer II (Qiagen, Hilden, Germany). DNA extractions were performed using Qiagen DNeasy Blood and Tissue Kit (Qiagen, Hilden, Germany) per manufacturer's instructions, using a final elution volume of 50 µl.

For quantification of the fungal pathogen load by quantitative real-time PCR, we designed primers based on GenBank sequence AY755505.1 to bind to the *Metarhizium brunneum* ITS2 rRNA gene region (Met-ITS2-F: 5′- CCCTGTGGACTTGGTGTTG-3′, Met-ITS2-R: 5′- GCTCCTGTTGCGAGTGTTTT-3′). Reactions were performed in 20 µl volumes using KAPA SYBR® FAST Bio-Rad iCycler 2X qPCR Master Mix (Kapa Biosystems), 3 pmol each of both primer (Sigma-Aldrich), and 2 µl of DNA template. The PCR program used for amplification was 95°C for 5 min, followed by 40 cycles of 10 s at 95°C and 30 s at 64°C. Each sample was run in triplicate. Each run included a negative control. Concentrations were determined using the standard curve method. Standards were obtained by extracting DNA of pure *Metarhizium* spores. The dilution series for the standard curve spanned the following DNA concentration range: 1 ng µl^−1^ to 1 × 10^−6^ ng µl^−1^. Specificity was confirmed by performing a melting curve analysis after each run.

Four control queens were killed by freezing 24 days after the treatment to exclude fungal infection. Similarly, nine queens treated with *M. brunneum* spores were killed to quantify their fungal pathogen load. To verify actual infection in our experiment and to exclude external contamination by attached conidiospores, we determined the amount of fungus on the ant's cuticle directly after exposure. To do so we exposed nine additional queens from stock colonies as described above and after 10 min killed them by freezing. We determined the value of this exposure dose by the same method of quantitative real-time PCR, and used it as a baseline to determine if the pathogen load increased as compared to this initial exposure dose, or not (see electronic supplementary material, S1). Only successful infection and pathogen replication inside the host body can lead to higher values in the experimental queens than in the exposure baseline. Queens with a higher than baseline value were thus categorized as highly infected (‘High infection’). Queens exposed to *M. brunneum* but showing pathogen loads below the exposure baseline, but above the negative control threshold, were categorized as having a low-level infection (‘Low infection’), so that exposed queens were separated into a high and low infection group according to their pathogen load. Only 19 of the originally 31 exposed queens could be used in the analysis (High infection *n* = 10; median age at treatment: 65 days; Q1: 60.8; Q3: 69.3), Low infection *n* = 9; median age at treatment: 67 days; Q1: 60; Q3: 70), as several queens did not survive the treatment procedure, did not resume egg laying, or their corpses could not be retrieved. For the same reasons, only six of 12 originally set up control queens (Triton X + untreated control group) could be used in the analysis, so that we pooled the two control treatments (Triton X, *n* = 3; Untreated, *n* = 3) to a single control group (*n* = 6 queens; median age at treatment: 63 days; Q1: 54.8; Q3: 71.3), as egg laying did not differ between them (Wilcoxon rank-sum test: before treatment *W* = 84.5, *p* = 0.5; after treatment *W* = 223, *p* = 1).

As the weekly reproduction rate of *C. obscurior* queens increases with lifespan [25], we compared individual egg-laying rates (daily egg number) during the week before (Before Treatment, BT, four scans; every second day) and the week after the treatment (After Treatment, AT, seven scans; daily). Data were analysed using R v. 3.2.3 [39] using packages ‘ggplot2’ [40] for all graphs (boxplots and LOESS curve) and ‘survival’ [41] for the Kaplan–Meier (KM) survival analysis and graph. Lifespans of queens, that had not died during the experimental period of 24 days and had to be frozen for qPCR analysis, were included as censored data in the survival analysis. Egg numbers before and after the treatment were not normally distributed (Shapiro–Wilk test, *W* = 0.97, *p* < 0.0001 and quantile–quantile plot analysis). Therefore, Kruskal–Wallis test was used for group comparisons followed by a pairwise Wilcoxon rank-sum test as *post hoc* test. We used Wilcoxon signed-rank test (paired) for two-sample comparisons. *p*-Values were adjusted using the Benjamini–Hochberg correction to protect against a false discovery rate of 5% in the library ‘fdr’ [42].

## 3. Results

**Figure 1.**
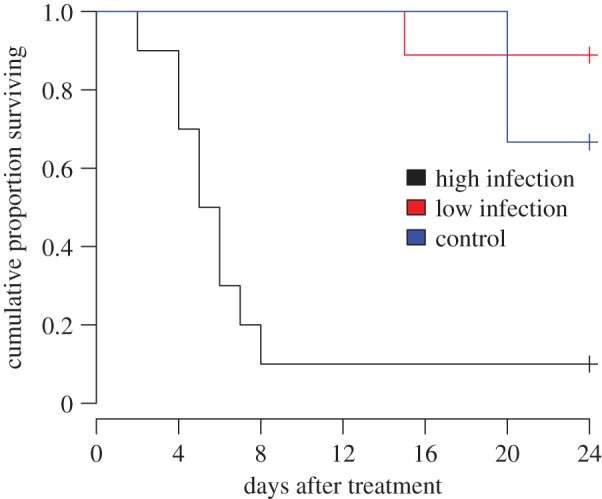
Survival of 41–74 day old *Cardiocondyla obscurior* queens was significantly decreased in queens suffering a high infection of the entomopathogenic fungus *Metarhizium brunneum*, as compared to both queens with a low infection and healthy control queens. The experiment was terminated 24 days after treatment and the lifespan of surviving queens were included as censored.

Of the 19 queens treated with the *Metarhizium brunneum* spore suspension that had been considered for analysis, nine (47.4%) died within 7 days after treatment. All surviving queens were killed after 24 days to check their infection status. Survival time was strongly influenced by infection level. Nine of 10 highly infected queens, but only one of nine lowly infected and two of six control queens died within 24 days after treatment. Highly infected queens thus died significantly earlier than control queens and queens with low infection (Survival analysis (KM): *χ*^2^ = 21, d.f. = 2, *p* < 0.0001, High infection, mean survival time ± s.d.: 7.1 ± 6.2 days, range 2–24 days; Low infection, mean survival time 23 ± 3 days, range 15–24 days; Control, mean survival time 22.7 ± 2 days, range 20–24 days, figure 1). The survival time after the treatment was not correlated with the age at treatment (Spearman's rank correlation: *ρ* = −0.005; *S* = 2613.2; *p* = 0.98).

**Figure 2.**
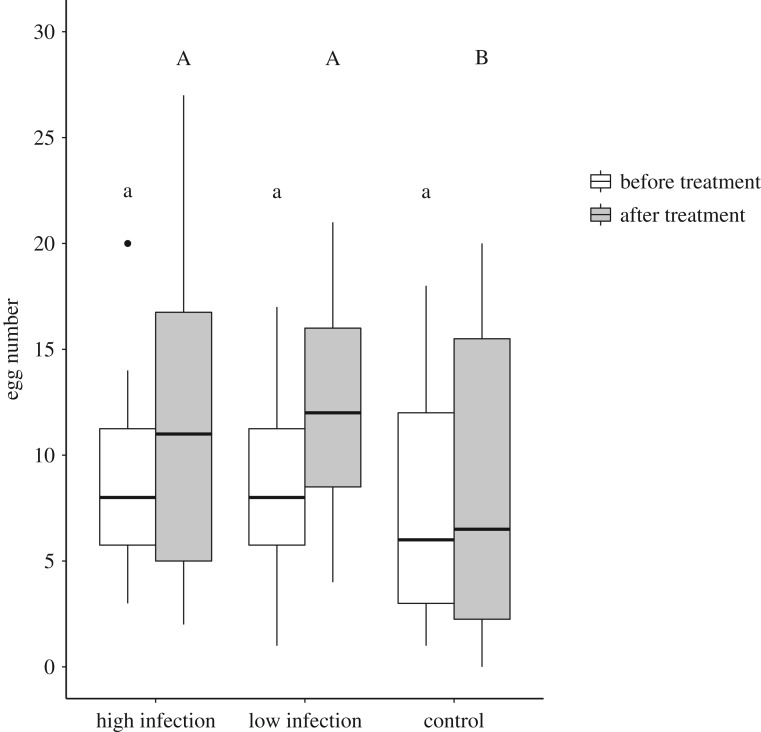
Egg number laid by *Cardiocondyla obscurior* queens in the week before and after treatment. Queens infected with *Metarhizium brunneum*, independent of their infection level (High or Low infection) laid significantly more eggs than Control queens, after—but not before—treatment. Boxplots show median, 25 and 75 quartile and 95% percentiles. Significantly different *post hoc* comparisons among groups are displayed by different letters (lower case before treatment, capitals after treatment), using an *α* = 0.05 of the Benjamini–Hochberg adjusted *p* values.

Egg-laying rates (daily egg number) did not differ among the three groups before the treatment (Kruskal–Wallis test: *χ*^2^ = 1.1, d.f. = 2, *p* = 0.57, figure 2). However, treatment affected egg-laying rates: independent of infection level, infected queens significantly increased their egg-laying rate after treatment relative to that before treatment (High infection: increase in egg-laying rate 1.3 times; Wilcoxon signed-rank test: *V* = 104.5, *p* = 0.0005; low infection: increase in egg-laying rate 1.5 times; *V* = 73, *p* = 0.0001, table 1). In contrast, weekly reproductive rate did not change in the control group (*V* = 66; *p* = 0.25).

**Table 1.** Eggs laid by 41–74 day old *Cardiocondyla obscurior* queens in the week before and after exposure with the entomopathogenic fungus *Metarhizium brunneum.*

eggs per week	high infection	low infection	control
before treatment			
median, quartiles	8.0 [5.8, 11.3]	8.0 [5.8, 11.3]	6.0 [3.0, 12.0]
after treatment			
median, quartiles	11.0 [5.0, 16.8]	12.0 [8.5, 16.0]	6.5 [2.3, 15.5]

In none of the groups did egg-laying rate differ between the day before and the day after the isolation (Wilcoxon signed-rank test: Low infection: *V* = 2, *p* = 0.15; High infection: *V* = 7, *p* = 0.21; Control: *V* = 3, *p* = 0.60), suggesting that the manipulation with forceps and subsequent isolation did not have a negative effect on reproduction. As infection only occurs approximately 48 h after exposure, changes in egg-laying rate can thus be related to changes in infection state, independent of the handling procedure.

**Figure 3.**
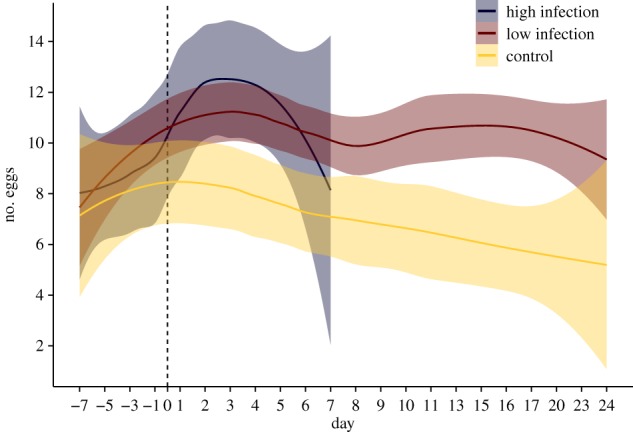
Temporal change in egg-laying rate (displayed by LOESS curve with 95% confidence intervals) for the week before treatment and the entire experimental time after the treatment. All but one highly infected queens had died within the first 7 days, so their curve is stopped after one week. While egg laying does not differ between the three groups before treatment, infected queens of both the high infection (blue) and low infection group (red) show increased egg laying over the control group (yellow).

Egg-laying rate in the week after treatment did not differ between highly and lowly infected queens, but both produced more eggs than the control group (Kruskal–Wallis test: *χ*^2^ = 10.5, d.f. = 2, *p* = 0.005; Pairwise Wilcoxon rank-sum test: High versus Control; *p* = 0.013; Control versus Low *p* = 0.007; High versus Low, *p* = 0.73, Benjamini–Hochberg corrected *p*-values). Queens with a low infection continued to lay more eggs than control queens throughout the remaining experimental time (Wilcoxon rank-sum test day 8–24, *W* = 3788, *p* < 0.0001, figure 3), while all except one highly infected queen had died by then. Infection load of queens treated with *M. brunneum* spores (estimated by qPCR) and mean egg number per queen were not correlated (Spearman's rank correlation: *ρ* = 0.08, *p* = 0.74; for infection loads see electronic supplementary material, table S1).

## 4. Discussion

The short lifespan of queens of the ant *Cardiocondyla obscurior* makes them a useful model to study the interrelation among life-history traits in social insects. We have previously shown that the fecundity of queens of *C. obscurior* increases gradually with age [25] and that they are capable of adjusting their egg-laying rate to changing social or environmental conditions without a reduction of lifespan [43,44]. Here we document that queens infected with an entomopathogenic fungus increase their egg-laying rate compared to uninfected queens of the same age. *Metarhizium brunneum* occurs in many parts of the world, e.g. in Europe [45] and Central America [32], and an infestation with this and other pathogens might be a permanent threat for ant colonies. Almost half of all exposed queens (47.3%) of *C. obscurior* died within one week after spore contact. An infection with *M. brunneum* is therefore associated with a severely reduced lifespan and the capability of queens to increase their reproductive efforts in response to infection is in line with the predictions of the terminal investment hypothesis [4], which has not been previously tested in social insects.

Fungal exposure did not always lead to lethal high-level infection. However, the behavioural and immunological defences of the ants led to asymptomatic low-level infections in 47% of the exposed queens, which did not induce mortality during the experimental duration of 24 days. Here we can show that also these low-level infected queens showed an increased egg-laying rate. This suggests that the reduction of lifespan in highly infected queens was not caused by a reinforced reproduction but by the infection. Low-level infections, as well as non-pathogenic injury, such as the experimental amputation of parts of a queen's leg, cause immune reactions [35,46]. Both these treatments do not have immediate lethal effects, but may still have later effects on the queens. These could not be examined in this study, given its limited experimental time of 24 days to determine their infection status.

In contrast to a pathogenic threat, non-infectious wounding induces a strong, but transient decrease of the egg-laying rate [46]. This indicates that infection and injury can have opposite effects on fecundity, despite both involving an immune response. The wounding study reveals that amputation leads to a trade-off between costly wound repair and reproduction in *C. obscurior* queens, in line with a general trade-off between resource allocation for reproduction and life sustaining processes [46,47]. As egg-laying rate was only decreased temporarily and returned to the level before the injury [46], we suggest that the costs for recovery were only transient. A short increase of investment into the immune system might have accelerated the recovery process, so that queens could resume their normal egg-laying rate quickly. The fact that fungal infection increased egg-laying rate indicates that it did not trigger a costly immune response. In any case, the net result is increased fecundity, which may compensate at least in part for the prospective decrease of residual reproductive value. Queens of social insects are supplied with food by workers and hence are usually not resource-limited [48]. This might allow them to invest into both immune response and reproduction, if necessary, as queens with low infections seem to be able to reduce spore proliferation compared to highly infected queens and are nevertheless able to increase their egg-laying rate.

Interestingly, studies on social insect workers showed a different response to an immune challenge. Rather than staying in the nest, infected or injured workers leave the nest and commence foraging earlier than unmanipulated nest-mates [49–51] and later die away from their natal colony [38,52,53]. Similarly, CO_2_ narcosis affects queen and worker behaviour in opposite directions: it initiates egg laying in queens but inhibits ovary activation and causes precocious foraging and death in isolation in workers [38,52–55]. This suggests a contrarious, caste-specific regulation of the physiological and behavioural responses to stressors, such as pathogens, injuries, or CO_2_ (e.g. [54]).

In conclusion, in addition to the previously shown increase of fecundity with age, our results clearly show that queens are able to adjust their egg-laying rate after infection with an obligate-killing fungal pathogen that induces queen mortality if causing high-level infection. Hence, our study strongly corresponds to the predictions of the terminal investment hypothesis.

## Supplementary Material

S1_Pathogen load

## Supplementary Material

Raw data

## Data accessibility

The datasets supporting this article have been uploaded as part of the electronic supplementary material.
