# Supplementary material for: Ant queens increase their reproductive efforts after pathogen infection
Source: R Soc Open Sci. 2017 Jul 5;4(7):170547. doi: 10.1098/rsos.170547 (PMC5541571; doi:10.1098/rsos.170547)
Supplement: S1_Pathogen load [file rsos170547supp1.docx]

Supplementary Material

Table S1, Infection loads of all *Cardiocondyla obscurior* queens either treated with *M. brunneum* (Met), 0.05% Triton X (Tx) or left untreated (Co). Highly infected queens are marked in grey.

| Sample | Cq Mean | Cq Std. Dev | SQ Mean | SQ Std. Dev | Treatment |
| --- | --- | --- | --- | --- | --- |
| 3 | 31,56 | 0,531 | 1,67E-06 | 5,55E-07 | Co |
| 6 | 31,50 | 0,825 | 2,56E-06 | 1,21E-06 | Co |
| 7 | 31,54 | 0,338 | 2,33E-06 | 5,58E-07 | Co |
| 106 | 15,48 | 0,024 | 1,54E-01 | 2,57E-03 | Met |
| 108 | 30,83 | 0,430 | 2,77E-06 | 7,67E-07 | Met |
| 111 | 21,08 | 0,086 | 2,84E-03 | 1,76E-04 | Met |
| 114 | 16,47 | 0,048 | 7,61E-02 | 2,60E-03 | Met |
| 117 | 17,73 | 0,076 | 2,58E-02 | 1,32E-03 | Met |
| 12 | 22,28 | 0,090 | 1,21E-03 | 7,63E-05 | Met |
| 24 | 29,17 | 0,014 | 1,13E-05 | 1,08E-07 | Met |
| 26 | 31,12 | 0,579 | 2,32E-06 | 8,34E-07 | Met |
| 30 | 31,44 | 0,719 | 1,88E-06 | 8,02E-07 | Met |
| 31 | 28,57 | 0,265 | 1,37E-05 | 2,71E-06 | Met |
| 40 | 30,06 | 0,184 | 4,70E-06 | 6,39E-07 | Met |
| 45 | 30,96 | 0,953 | 2,80E-06 | 1,52E-06 | Met |
| 52 | 31,74 | 0,477 | 1,46E-06 | 4,55E-07 | Met |
| 54 | 18,36 | 0,059 | 1,97E-02 | 8,28E-04 | Met |
| 60 | 29,96 | 0,164 | 6,66E-06 | 7,21E-07 | Met |
| 83 | 31,24 | 0,121 | 2,03E-06 | 1,71E-07 | Met |
| 88 | 17,98 | 0,136 | 2,59E-02 | 2,58E-03 | Met |
| 90 | 32,12 | 0,367 | 1,10E-06 | 3,03E-07 | Met |
| O | 14,84 | 0,111 | 1,83E-01 | 1,37E-02 | Met |
| 61 | 0,00 | 0,000 | 0,00E+00 | 0,00E+00 | Tx |
| 64 | 31,10 | 0,391 | 2,29E-06 | 6,67E-07 | Tx |
| 65 | 31,24 | 0,573 | 2,13E-06 | 9,32E-07 | Tx |
